# A randomized phase 3 trial of total neoadjuvant therapy (induction chemotherapy, neoadjuvant chemoradiation, neoadjuvant chemotherapy, and surgery) vs. standard long-term chemoradiation therapy (neoadjuvant chemoradiation, surgery, and adjuvant chemotherapy) in locally advanced rectal cancer

**DOI:** 10.3389/fonc.2024.1468279

**Published:** 2024-12-05

**Authors:** Freshte Foroughi, Seyed Alireza Javadinia, Roham Salek

**Affiliations:** ^1^ Student Research Committee, Mashhad University of Medical Sciences, Mashhad, Iran; ^2^ Non-Communicable Diseases Research Center, Sabzevar University of Medical Sciences, Sabzevar, Iran; ^3^ Cancer Research Center, Mashhad University of Medical Sciences, Mashhad, Iran

**Keywords:** rectal cancer, total neoadjuvant therapy, neoadjuvant chemotherapy, adjuvant chemotherapy, neoadjuvant chemoradiotherapy, randomized controlled trial

## Abstract

**Purpose:**

The management of rectal adenocarcinoma has evolved during the last decade, shifting from a conventional neoadjuvant chemoradiotherapy, surgery, and adjuvant chemotherapy in all cases to a total neoadjuvant approach, especially in locally advanced tumors when a sphincter-sparing surgery has been planned. However, the exact indications and the neoadjuvant regimen with the highest response remain unresolved. We aimed to assess whether administering neoadjuvant chemotherapy before and after preoperative chemoradiotherapy could increase the pathological complete response (pCR) rates.

**Methods:**

We conducted a phase 3, multicenter, randomized trial at four hospitals in Iran. Adult patients with a newly diagnosed, biopsy-proven, locally advanced non-metastatic rectal adenocarcinoma with an ECOG performance status of 0–2 were randomly assigned (2:2) to either the total neoadjuvant treatment (TNT) or the standard-of-care groups using a block randomized design. Investigators and participants were not masked to treatment allocation and groups. The TNT group received neoadjuvant chemotherapy with FOLFOX6 (intravenous 85 mg/m^2^ oxaliplatin and 400 mg/m^2^ leucovorin, followed by intravenous 400 mg/m^2^ fluorouracil bolus and then continuous infusion at a dose of 2,400 mg/m^2^ over 46 h every 14 days for four cycles before and four cycles after chemoradiotherapy), chemoradiotherapy (50.4 Gy during 28 fractions and 800 mg/m^2^ concurrent oral capecitabine twice daily 5 days per week), and total mesorectal excision. The standard-of-care group received neoadjuvant chemoradiotherapy, total mesorectal excision, and adjuvant chemotherapy (eight cycles). The primary endpoint was the pathological complete response. Safety analyses were conducted on treated patients.

**Results:**

Overall, 25 and 27 patients were enrolled in the TNT and standard-of-care groups, respectively. Both groups were similar in terms of gender, age, and tumor differentiation. The tumors in the standard-of-care group were significantly located closer to the anal verge compared with those in the TNT group (9.4 ± 3.7 cm in TNT vs. 6.8 ± 4 cm in standard, *p* = 0.02). A pCR was reached in 48% (12/25) and 25.9% (7/27) of patients in the TNT and standard-of-care groups, respectively (*p* = 0.4). The R0 resection rates were identical between the two groups (92% vs. 88.9%, *p* = 0.3). Moreover, the toxicity rates were similar between the two groups.

**Conclusion:**

Our results showed that TNT is a safe and feasible treatment approach in patients with rectal cancer and may improve the overall pCR rate compared with standard treatment.

**Clinical trial registration:**

https://irct.behdasht.gov.ir/trial/65666, identifier IRCT20220723055527N1.

## 1 Introduction

Rectal cancer, as one of the most prevalent malignancies of the gastrointestinal tract worldwide, is diagnosed in advanced disease in almost half of patients. These tumors are defined as any node-positive cancers or any cancers with local invasion to surrounding tissues (1). Considering the increasing trends in the diagnosis and mortality rates of rectal cancer in younger patients (2), it is essential to maintain the quality of life of patients by preserving the intestinal integrity and avoiding permanent colostomy while prescribing the most efficient treatment approach by decreasing the probability of locoregional recurrence and distant metastasis (3). To achieve these goals, there has been a shift from neoadjuvant chemoradiotherapy (CRT), surgery, and adjuvant chemotherapy as conventional treatment approaches for rectal cancer to total neoadjuvant therapy (TNT) to prescribe all of the non-surgical medical modalities before surgery (4–7).

Due to limited resources and the long waiting lists for radiotherapy, it is a common practice in developing countries to prescribe a number of chemotherapy cycles before neoadjuvant CRT (8, 9). However, there has been no randomized trial assessing this treatment approach in this situation. In this prospective randomized trial, we aimed to assess whether administering neoadjuvant chemotherapy before and after preoperative CRT could increase the pathological complete response (pCR) rate.

## 2 Methods

### 2.1 Participants

The study was conducted at four main tertiary referral cancer treatment centers in the northeast of Iran, i.e., the Oncology Clinics of Imam Reza and Omid Educational Hospitals, both affiliated with Mashhad University of Medical Sciences, Reza Radiotherapy Oncology Center, affiliated with Mashhad Cancer Charity, and Vasei Educational Hospital, affiliated with Sabzevar University of Medical Sciences, between November 2022 and July 2024.

We enrolled newly diagnosed adult patients with biopsy-proven, locally advanced non-metastatic rectal adenocarcinoma who had an ECOG performance status of 0–2, normal kidney (creatinine <1.6 mg/dL) and liver functions [serum glutamic–pyruvic transaminase (SGPT) and serum glutamic–oxaloacetic transaminase (SGOT) less than three times the upper limit of normal] tests, and adequate bone marrow capacity (hemoglobin levels higher than 10 g/mL, absolute neutrophil count higher than 1.9 × 10^9^/L, and platelet count higher than 100 × 10^9^/L).

Patients were excluded in cases with a previous history of malignancy rather than skin basal cell carcinoma, previous treatment with chemotherapy and/or radiotherapy, presence of synchronous or metachronous primary cancers at different sites of the gastrointestinal tract, distant metastasis at diagnosis, history of inflammatory bowel disease, history of bilateral total hip replacement, dihydropyrimidine dehydrogenase deficiency, and oxaliplatin-induced hypersensitivity reactions.

### 2.2 Study design

In this randomized, parallel-group trial, adult patients with a newly diagnosed, biopsy-proven, locally advanced non-metastatic rectal adenocarcinoma with an ECOG performance status of 0–2 were randomly assigned (2:2) to either the total neoadjuvant treatment (TNT) group or the standard-of-care group using a block randomized design. In this context, the letter A or B was allocated to the six-cycle or the 12-cycle group, respectively, drawing four potential combinations (i.e., AABB, BBAA, ABAB, and BABA). The envelope randomization method was used to assign patients to each group. The investigators and the participants were not masked to treatment allocation and treatment groups.

Before starting the treatment, all patients underwent computed tomography (CT) scan of the chest, abdomen, and pelvis to assess the presence of distant metastasis and pelvis magnetic resonance imaging (MRI) scan to assess the local extension of the tumor. Moreover, complete blood count (CBC), kidney function test (KFT), liver function test (LFT), and carcinoembryonic antigen (CEA) and carbohydrate antigen 19-9 (CA 19-9) tests were conducted. A staging workup according to the latest version of the National Comprehensive Cancer Network (NCCN) guidelines for Rectal Cancers (10–12) was also performed. The TNT group received neoadjuvant chemotherapy with FOLFOX6 (intravenous 85 mg/m^2^ oxaliplatin and 400 mg/m^2^ leucovorin, followed by intravenous 400 mg/m^2^ fluorouracil bolus and then continuous infusion at a dose of 2,400 mg/m^2^ over 46 h every 14 days for four cycles before and four cycles after CRT) (13), CRT (50.4 Gy during 28 fractions and 825 mg/m^2^ concurrent oral capecitabine twice daily 5 days per week) (14), and total mesorectal excision. The standard-of-care group received neoadjuvant CRT, total mesorectal excision, and adjuvant chemotherapy (eight cycles). After completion of the neoadjuvant treatments, patients underwent surgery within 4–6 weeks.

It is worth mentioning that, based on the initial protocol, the trial was designed to prescribe the whole course of neoadjuvant chemotherapy after preoperative chemoradiation. However, due to the long waiting lists for radiotherapy, the trial design was changed in order to prescribe four cycles of neoadjuvant chemotherapy before the preoperative chemoradiation and four cycles after it.

Before each course of chemotherapy, the patients were asked about their signs and symptoms, and they underwent physical examination and CBC to assess the treatment toxicity. During the chemoradiation, the patients were visited weekly to assess toxicities. After completion of treatments and the surgery, the patients were followed up regarding the pathological response to the neoadjuvant treatments, with CEA and CA 19-9 measurements every 3 months, annual CT scans of the chest, abdomen, and pelvis, and colonoscopy every 5 years to detect local recurrence and distant metastasis.

### 2.3 Variables

The primary endpoint was the pathological complete response. The secondary endpoint was the sphincter-preserving surgery rate. Safety analyses were performed on treated patients.

#### 2.3.1 Pathological response to neoadjuvant treatments

To evaluate the response of tumor to neoadjuvant treatments, the Tumor Regression Grading (TRG) classification was adopted (15). Accordingly, TRG 0–3 were categorized as no residual tumor cells, presence of single cells or small groups of cells, presence of residual cancer with desmoplastic response, and minimal evidence of tumor response (16, 17).

Moreover, the residual tumor (R) classification was reported to describe the absence or presence of residual tumor following the surgery: R0, no residual tumor; R1, microscopic residual tumor; and R2, macroscopic residual tumor (18, 19).

#### 2.3.2 Sphincter-preserving surgery rate

All patients were assessed regarding whether they underwent permanent stoma or sphincter-preserving resection. If a patient received diverting colostomy, he/she was followed up to determine the final status of the sphincter.

#### 2.3.3 Treatment toxicity

The Eastern Cooperative Oncology Group Common Toxicity Criteria V.5.0 (ECOG-CTC) was used to assess chemotherapy-induced neutropenia, thrombocytopenia, anemia, constipation, diarrhea, nausea, vomiting, and alopecia using a four-grade scoring system (from the mildest to the most severe, grade 0 to grade 4).

Moreover, patients were followed up to 1 month to assess complications associated with surgery, including delayed wound healing, fistula, intra-abdominal infection/abscess, obstruction, and surgical scar infection.

### 2.4 Ethics

This trial was registered in the Iranian Registration of clinical trials (IRCT20220723055527N1), prospectively.

The study protocol was approved by the Ethics Committee of Mashhad University of Medical Sciences (approval code: IR.MUMS.fm.REC.1396.449) and Sabzevar University of Medical Sciences (IR.MEDSAB.REC.1402.089) and was conducted according to the Declaration of Helsinki.

Undersigned informed consent forms were obtained from all patients prior to enrollment.

### 2.5 Statistical analyses and sample size

#### 2.5.1 Sample size

Considering tumor regression rates of 75% and 41% in patients with rectal cancer receiving TNT and those in the standard-of-care group, respectively (20), with a type I error rate of 0.05 and a statistical power of 80%, the sample size was calculated to be 30 patients per group $\left(n=\frac{{\left(Z_{1-\frac{\alpha }{2\;}}+Z_{1-\beta }\right)}^{2}\left(P_{1}\left(1-P_{1}\right)+P_{2}\left(1-P_{2}\right)\right)}{{\left(d\right)}^{2}}\right)$ . However, due to potential loss to follow-up, we designed the trial to enroll at least 50 patients in each group.

#### 2.5.2 Statistical analyses

Normality of the data was assessed with the Shapiro–Wilk test using the Statistical Package for the Social Sciences version 22 (SPSS Inc., Chicago, IL, USA). All data had normal distribution. Therefore, the categorical and quantitative data were analyzed using a chi-square test (Fisher’s exact test) and a *t*-test, respectively. Intention-to-treat analysis was adopted to perform statistical analysis. Survival data were presented using Kaplan–Meier curves and were analyzed using univariate log-rank. A *p*-value <0.05 was considered statistically significant.

## 3 Results

### 3.1 Patients

**Figure 1:**
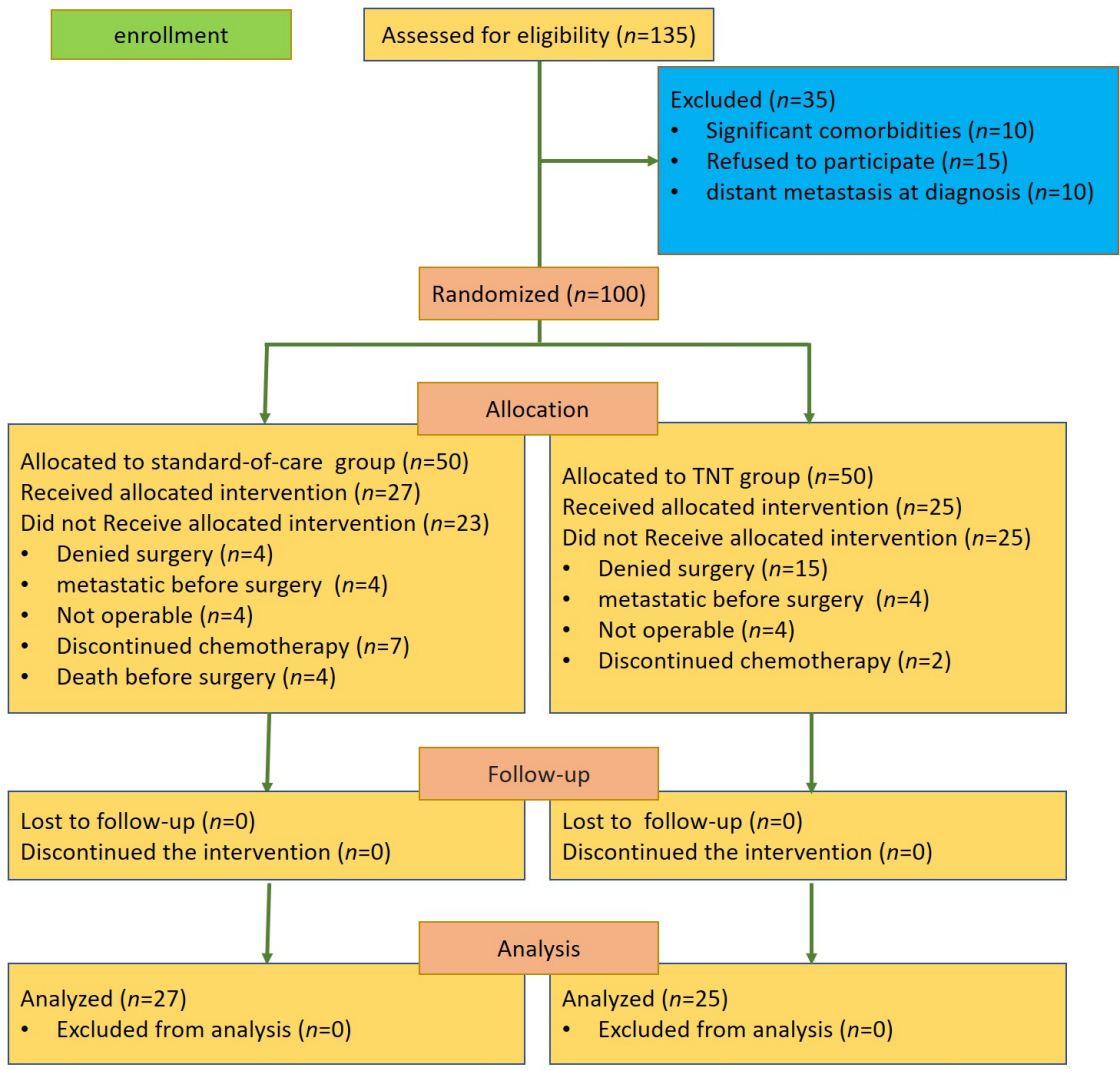
Consort flow diagram.

From November 2022 to July 2024, a total of 100 patients from four institutions in Mashhad and Sabzevar, Iran, were randomly assigned into the TNT group or the standard-of-care group. In the TNT group, 15 patients denied surgery, four patients were diagnosed with distant metastases before the surgery, four patients were considered non-operable, and two patients discontinued neoadjuvant chemotherapy. In the standard-of-care group, four patients denied surgery, four patients were diagnosed with distant metastases before the surgery, four patients died before the surgery, four patients were considered non-operable, and seven patients discontinued adjuvant chemotherapy (**Figure 1**).

Both groups were similar in terms of gender, age, and tumor differentiation. The tumors in the standard-of-care group were significantly located closer to the anal verge compared with those in the TNT group (9.4 ± 3.7 cm in TNT *vs*. 6.8 ± 4 cm in standard, *p* = 0.02) (**Table 1**).

**Table 1:** Characteristics of the patients at baseline.

Characteristics	Entire study: 52 patients		*p*-value
	TNT: 25 patients *n* (%)	Standard: 27 patients *n* (%)	
Male gender	16 (64)	17 (63)	0.9
Age (mean ± SD)	54.3 ± 11.9	59.2 ± 9.8	0.1
Tumor differentiation			
Well differentiated	15 (60)	12 (44.4)	0.4
Moderately differentiated	7 (28)	12 (44.4)	
Poorly differentiated	3 (12)	3 (11.1)	
Tumor location			
0–5 cm from the anal verge	4 (16)	13 (48.1)	0.01
5.1–10 cm from the anal verge	12 (48)	12 (44.4)	
10.1–15 cm from the anal verge	9 (36)	2 (7.4)	
Distance from anal verge (cm)	9.4 ± 3.7	6.8 ± 4	0.02

TNT, total neoadjuvant treatment.

### 3.2 Pathological response rates

**Figure 2:**
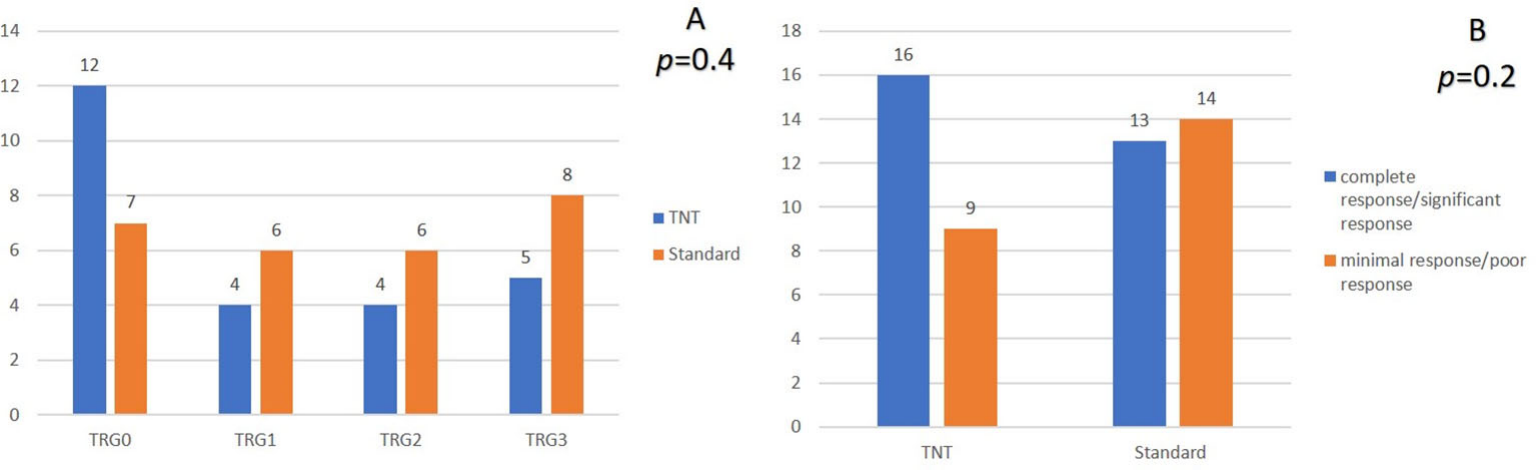
Comparison of the pathological response in the two groups. **(A)** comparison based on TRG groups. **(B)** comparison after reclassification TRG groups. TRG, Tumor regression grade.

A pCR was reached in 48% (12/25) and in 25.9% (7/27) of patients in the TNT group and the standard-of-care group, respectively (*p* = 0.4) (**Figure 2A**). After categorizing the responses, pathological complete and near-complete responses were reported in 64% (16/25) of patients in the TNT group, which was insignificantly higher than that of the patients in the standard-of-care group (48.1%, *p* = 0.2) (**Figure 2B**). The R0 resection rates were also identical between the two groups (92% vs. 88.9%, *p* = 0.3) (**Table 2**).

**Table 2:** Comparison of the R resection in the two groups.

Characteristics	Entire study: 52 patients		*p*-value
	TNT: 25 patients *n* (%)	Standard: 27 patients *n* (%)	
R resection			
R0	23 (92)	24 (88.9)	0.3
R1	1 (4)	3 (11.1)	
R2	1 (4)	0	

TNT, total neoadjuvant treatment.

### 3.3 Sphincter-preservation rates

Sphincter-preserving surgery was performed in 60% of patients in the TNT group and in 77.8% of patients in the standard-of-care group (*p* = 0.1) (**Table 3**).

**Table 3:** Comparison of the sphincter-preservation rates in the two groups.

Characteristics	Entire study: 52 patients		*p*-value
	TNT: 25 patients *n* (%)	Standard: 27 patients *n* (%)	
Sphincter-preserving surgery			
No	10 (40)	6 (22.2)	0.1
Yes	15 (60)	21 (77.8)	

TNT, total neoadjuvant treatment.

### 3.4 Safety

Adjustment of the chemotherapy dose during radiotherapy was reported in 12% of patients in the TNT group, while no dose adjustment was reported in the standard-of-care group (*p* = 0.06). Unscheduled gaps in radiotherapy treatment were reported in 8% (2/25) and 7.4% (2/27) of patients in the TNT and standard-of-care groups, respectively (*p* = 0.9). Moreover, adjustment of the chemotherapy dose during neoadjuvant/adjuvant treatments was reported in 16% (*n* = 4/25) and in 14.8% (*n* = 4/27) of patients in the TNT and standard-of-care groups, respectively (*p* = 0.9). Treatment gaps during neoadjuvant/adjuvant chemotherapy were reported in 16% (4/25) of patients in the TNT group and in 18.5% (5/27) of patients in the standard-of-care group. Data on toxicities are illustrated in **Table 4**.

**Table 4:** Treatment toxicity in patients in the two groups.

Adverse event	TNT: 25 patients *n* (%)				Standard: 27 patients *n* (%)			
	G1	G2	G3	G4	G1	G2	G3	G4
Chemoradiotherapy								
Neutropenia	0	0	0	0	0	0	0	0
Thrombocytopenia	2 (8)	0	0	0	3 (11.1)	0	0	0
Anemia	2 (8)	0	0	0	3 (11.1)	0	0	0
Tenesmus	0	0	0	0	1 (3.7)	0	0	0
Diarrhea*	7 (28)	0	0	0	1 (3.7)	0	0	0
Dysuria	2 (8)	0	0	0	1 (3.7)	0	0	0
Chemoradiotherapy								
Anorexia	0	0	0	0	1 (3.7)	0	0	0
Nausea/vomiting	2 (8)	0	0	0	1 (3.7)	0	0	0
Anemia	1 (4)	0	0	0	1 (3.7)	0	0	0
Thrombocytopenia	2 (8)	0	0	0	0	0	0	0
Neutropenia	2 (8)	1 (4)	0	0	2 (7.4)	0	0	0
Diarrhea	2 (8)	0	0	0	2 (7.4)	0	0	0
Surgery								
Impaired wound healing	1 (1)	0	0	0	0	0	0	0
Fistula	0	0	0	0	0	0	0	0
Intra-abdominal infection/abscess	0	0	0	0	0	0	0	0
Obstruction	0	0	0	0	1 (3.7)	0	0	0

TNT, total neoadjuvant treatment.

*p = 0.01.

## 4 Discussion

Our results indicated that, compared with neoadjuvant CRT, the use of a TNT approach could enhance the response to neoadjuvant treatments, although the difference was not significant. On the other hand, the sphincter-preservation rates were not significantly lower in the TNT group while the tumors were located further away from the anal verge. With regard to the safety profile, both the TNT and neoadjuvant CRT approaches were well-tolerated, and most of the toxicities were grades I and II.

This study showed that TNT doubled the pCR rate compared with neoadjuvant chemoradiation (from 25.9% to 48%). This finding is similar to the pCR rates reported in recently published trials [the STELLAR trials (22% vs. 12%), the UNICANCER-PRODIGE 23 (28% *vs*. 12%), and the RAPIDO (28% vs. 14%)] (4, 5, 21). It is evident that prescription of all treatment modalities before surgery in patients with rectal cancer is an independent factor for inducing a pCR (22). The higher pCR rates following the TNT approach could be due to the intact cellular and extracellular components of the microenvironment and better oxygenation of the tumoral lesion before the surgery, promoting synergetic effects of the cytotoxic agents and radiotherapy and a longer interval time before the surgery to induce the cytotoxic effects (23). The pCR rates reported in our study were considerably higher than those reported in the above-mentioned trials. While our approach in the TNT group consisted of a neoadjuvant long-course radiotherapy with concomitant chemotherapy and neoadjuvant FOLFOX6, the RAPIDO and the STELLAR trials applied a short-term radiotherapy in the TNT group. Moreover, in the STELLAR trial, the patients in the TNT group only received four cycles of CAPOX (oxaliplatin and capecitabine). In addition, in the UNICANCER-PRODIGE 23 trial, the patients received six cycles of FOLFIRINOX (leucovorin, fluorouracil, irinotecan, and oxaliplatin), which incorporated irinotecan into the preoperative treatment for rectal cancer. These differences in the applied approaches could be the reason for the differences in the pCR rates. In studies on rectal cancer in Iran by Joybari et al., Novin et al., and Aghili et al., the pCT was reported to be between 20% and 30% (8, 9, 24). In the small study by Joybari et al., 29 patients received two courses of FOLFOX4 before surgery (9). Moreover, in the study by Aghili et al., 32.3% of patients (51/400) received preoperative chemotherapy as an induction and/or a consolidation approach. It appears that the unique geographical composition and the diversity of the Iranian population may have influenced the responses to neoadjuvant treatments in patients with rectal cancer (25). Collectively, our results demonstrated that the post-treatment pCR rate in the TNT arm is nearly double that reported in the most prominent literature. This improvement may be attributed to the intensification of neoadjuvant treatment with eight cycles of FOLFOX (26). Furthermore, real-world applications of neoadjuvant treatments have shown similar results (27, 28). Moreover, the patients included in the present study received a standard radiation dose (50.4 Gy in 28 fractions). However, as previous studies have demonstrated, pCR can be influenced by the radiation dose. For example, when using simultaneous integrated boost (SIB) techniques, doses up to 55–60 Gy have been shown to improve the pCR rates (29, 30). Therefore, intensification of chemotherapy and radiotherapy, especially in the era of novel treatment strategies such as immunotherapy and intensity-modulated radiation therapy/volumetric-modulated arc therapy/image-guided radiotherapy (IMRT/VMAT/IGRT), should be taken into consideration in future efforts.

With regard to the resectability of tumor, while the R0 resection rates were similar between the TNT (92%) and standard-of-care (88.9%) groups, the number of patients not being operable following the neoadjuvant treatments regardless of the approach was considerable in both groups (4/50 patients). The rates are similar to those in previous reports showing high rates of R0 resections, with R0 resection rates of 81% for conventional neoadjuvant CRT, 86% for induction chemotherapy followed by neoadjuvant CRT, 86% for long-course CRT followed by consolidation chemotherapy, and, finally, 82% for modified short-course radiotherapy followed by consolidation chemotherapy (31, 32). It appears that there is a subgroup of patients who do not respond properly to the neoadjuvant treatments while not developing any distant metastases. Therefore, it is essential to use an accurate diagnostic tool to determine these patients and to intensify the treatments using novel targeted agents and immunotherapy drugs and new radiotherapy modalities in order to improve the local responses.

Consistent with previous findings, our results showed that most adverse events in both groups were tolerable, albeit adjustment of the chemotherapy dose, unscheduled gaps in radiotherapy treatment, and treatment gaps during neoadjuvant/adjuvant chemotherapy were reported in a proportion of enrolled patients in both groups. This observation displays the importance of exact follow-up of patients during the treatment course to detect any deleterious effects of treatments and to determine appropriate management.

Except for patients who developed metastatic disease before surgery, 15 patients in the standard-of-care group and 21 patients in the TNT group were excluded due to discontinued chemotherapy, refusal to undergo surgery, or death before surgery. These exclusions impacted the power of the present study by removing a considerable number of patients from the final analysis. The follow-up of patients in this study is essential to determine the effects of TNT on local recurrence and the distant metastasis rates.

Moreover, a significant difference in tumor location was observed between the two groups. This finding was surprising to us as well. While patient assignment was randomized, it is possible that the intention to enroll those with more advanced disease in the TNT group and the assumption that TNT would lead to increased sphincter preservation may have influenced patient selection. Furthermore, the trial design was modified during the course of the study due to the long waiting lists for radiotherapy. Originally, all neoadjuvant chemotherapy was to be administered after chemoradiation, but this was changed to a split regimen, with portions administered before and after chemoradiation. This mid-study change could have introduced bias and affected the interpretation of the results.

## 5 Conclusion

Our results showed that TNT is a safe and feasible treatment approach in patients with rectal cancer, and it may improve the overall pCR rate compared with standard treatment.

## Data Availability

The raw data supporting the conclusions of this article will be made available by the authors, without undue reservation.
